# Research on the impact of industrial robot application on the status of countries in manufacturing global value chains

**DOI:** 10.1371/journal.pone.0286842

**Published:** 2023-06-08

**Authors:** Wenhua Yuan, Weixiao Lu

**Affiliations:** 1 Institute of International Economy, University of International Business and Economics, Beijing, China; 2 Business School, Yangzhou University, Yangzhou, Jiangsu, China; University of Economics Ho Chi Minh City, VIET NAM

## Abstract

The fast growth in the installation of industrial robots has had a major impact on the comparative advantage of nations and the division of labor in global value chains in the era of smart manufacturing. Using various econometric models and panel data from 18 industries in 38 countries from 2000 to 2014, this paper empirically examines the impact of industrial robot applications on the status of countries in manufacturing global value chains and its mechanisms. The study demonstrates that industrial robot application can effectively improve the status of countries in manufacturing global value chains, and this improving effect is more obvious for developing countries and labor-intensive and technology-intensive industries. Mechanism testing shows that industrial robot application can effectively enhance the development level of highly skilled human capital and productive service industries, thereby improving the status of the manufacturing global value chain. This study provides a theoretical basis and policy reference for countries to enhance their status in the global value chain through industrial robot applications in the future.

## 1. Introduction

In the era of global economic integration, the new technological waves and industrial revolution have a significant impact not only on the employment market and enterprise productivity in related countries but also on the status of global value chains (GVC) due to changes in relative production costs and the degree of specialization. Industrial robotics is a crucial technology that is paving the way for the future. It enables the completion of production tasks and work links with high accuracy and high efficiency and over long periods in dangerous and harsh environments, such as those with high and ultracold temperatures and toxicity. Additionally, programmable industrial robots have high flexibility and mobility. They can be combined with CNC machining centers, cloud computing platforms, and automatic detection systems to form flexible production systems, expanding the scope of enterprise production product variety and promoting the intelligent upgrading of manufacturing, which is considered the critical factor in promoting the "next generation of the production revolution." Many countries, especially developed countries, such as the U.S., have taken action and proposed national strategic policies to expand the use of industrial robots. Such efforts include the Advanced Manufacturing Leadership Strategy, the EU Horizon 2020 Strategy, the German Industry 4.0 Strategy, the French New Industrial Strategy, the Indian National Artificial Intelligence Strategy, the Made in China 2025 Strategy, etc. The application of industrial robots has become a way for countries to rebuild their international competitive advantage.

However, the current research on the impact of industrial robot applications on the value chain position of the manufacturing industry does not yet go in depth, and there are significant differences within each country [1]. This phenomenon also raises questions about whether industrial robots can increase the competitiveness of manufacturing GVC in each nation and how industrial robots affect the GVC of manufacturing.

To solve this problem, we match the International Federation of Robotics (IFR) database, World Input‒Output Database (WIOD) database, UIBE GVC Indicators database, and World Development Indicators (WDI) database, construct robot density indicators for each country by year and industry, adopt various methods, such as the fixed effects model, instrumental variables method, and system GMM, to empirically test the impact of industrial robot applications on the status of countries in manufacturing GVC, explore the specific mechanisms of this impact, and further explore whether industrial robot applications can reduce the status gap between countries and achieve "limited catch-up" in the extended analysis.

This paper’s research significance and innovations are as follows. First, we expand the scope of industrial robotics research by extending the current focus on labor market impacts on international trade and GVC. Second, we combine the human‒machine collaboration represented by highly skilled human capital and the producer services represented by the export technological sophistication of producer services with industrial robot applications and explore how industrial robot applications affect manufacturing GVC, which provides a starting point for corresponding industrial policy formulation in an era when countries are competing to introduce industrial robots and develop intelligent manufacturing. This has vital practical significance. Third, by studying the heterogeneity of the impacts and limited catch-up in GVC, we further deepen the understanding of the impact of industrial robot applications on sustainable development.

## 2. Literature review

The IFR definition of industrial robots is mainly used internationally. That is, if the machine is an "automatically controlled, reprogrammable multipurpose manipulator programmable in three or more axes," then the machine is considered an industrial robot. The research literature related to this topic can be divided into the following three main streams.

First, from the perspective of relative cost and offshoring, industrial robot applications would reduce the participation and relative the status of countries in manufacturing GVC. The development of science and technology has made it cheaper and easier for industrial robots to replace labor, and the labor cost advantages of developing countries are being eroded [2]. Developed countries upstream of GVC take the lead in applying industrial robots, which will lead to the "value-added erosion" of countries downstream of GVC [3]. Robots will not bring long-term growth, and machine-human competition resulting from biased technological progress depresses average worker wages. Robotization impacts labor by increasing upstream and positive global value chain integration [4], which in turn reduces worker savings and social investment and ultimately hinders economic growth [5]. Industrial robot applications reduce the relative advantage of developing countries in traditionally labor-intensive manufacturing, making the advantage of low-cost labor less attractive, reducing offshoring activities in developed countries, and weakening technological connections in traditional global value chains [6], which will lead to the exclusion of developing countries from GVC and hinder their development [7]. The increased density of industrial robots would make multinational companies more efficient in domestic production than in production abroad [8], triggering the return and re-offshoring of production and weakening the overall importance of GVC [9].

Second, from the standpoint of productivity, the use of industrial robots would raise the level of involvement and relative standing of the manufacturing GVC. By altering the labor productivity of businesses and the total factor productivity of entire nations, industrial robots have a considerable impact on the global distribution of economic activity and the structure of GVC. Enterprises with more industrial robots have a lower share of production labor, a higher share of capital, and higher labor productivity [10], which can significantly improve their competitiveness and promote the upgrading of global value chain technology [11], but this may also lead to increasing productivity differences that exacerbate the superstar phenomenon [12]. The use of industrial robots enhances the digital capabilities of companies and the efficiency of resource allocation [13], and increasing the proportion of industrial robot applications in developing countries can lead to higher technical efficiency, better sales and higher value added, enhancing the status of GVC [14]. The use of industrial robots also significantly expands the distribution network of multinational companies’ headquarters and the value creation of their subsidiaries, effectively enhancing their overall global value chain position [15]. The application of industrial robots has brought about improvements in productivity, optimization of factor structures, and green technology innovation in the global manufacturing sector [16], thereby improving energy efficiency, reducing carbon intensity, and enhancing the position of GVC [17].

Third, from the standpoint of international trade, industrial robot applications would increase the participation and relative the status of countries in manufacturing GVC. Based on task-based and Ricardian models, it is known that the increased installation of industrial robots in northern countries reduces the production costs of enterprises, which leads to an increase in final product exports to southern countries [18]. The increase in the exports of final products from northern countries has led to an increase in the demand for intermediate products from southern countries, which leads to an increase in real wages and GVC participation for workers in both the north and the south. Industrial robot applications can increase export technological sophistication, enhancing sustainable corporate global management and technology transfer [19], and intelligent manufacturing represented by industrial robots is an effective way to upgrade the structure of export products [20]. Industrial robot applications lead to higher firm productivity and broader production categories, prompting firms to import and invest more in subsidiaries in low-income countries, which can enhance value chain participation in both the MNC’s home and trading countries [21]. It is worth noting that simply increasing the minimum density of robots would harm regional trade in East Asia. If countries can widely use network technology, reduce the cost of digital services trade, and integrate it into the production network to enhance the complementarity between them, industrial robots can improve the overall competitiveness and the status of countries in GVC of all regional countries [22].

In summary, the current literature on the impact of industrial robot applications on the status of countries in GVC, especially from the perspective of trade, has laid a theoretical foundation for our study, but some deficiencies still exist. First, existing research mainly focuses on overall national competitiveness or microenterprise productivity and less on the impact of trade on value added by country and industry, which is most closely linked to GVC. Second, the existing research focuses on the direct impact of industrial robot applications on the status of countries in GVC, ignoring the critical impact of other economic factors on their effectiveness and lacking in-depth research and straightforward elucidation of their impact mechanism.

## 3. Theoretical analysis and research hypothesis

### 3.1 Overall effect

First, from the standpoint of enterprises, industrial robots can work continuously and uninterruptedly with high accuracy and efficiency in complex and harsh environments, shortening the production cycle and improving production accuracy. Moreover, the enhancement of product quality and specialization expands enterprises’ production scale and improves their product added value and profitability. The integration of industrial robots with new technologies changes the production and operation modes of enterprises, enhances productive flexibility with flexible production systems, and helps reduce their production costs. Technological upgrading enhances the production capacity of enterprises and increases the variety of products that can be produced, which is conducive to enterprises obtaining more market share and strengthening the advantage of economies of scope [23].

Second, from the standpoint of the industry, industrial robot technology, as a basic technology of advanced artificial intelligence, has the spillover characteristics of infrastructure and can play the "leading goose" effect, driving the conversion and upgrading of the whole GVC in the direction of intelligence and digitalization and improving the overall value-added rate of the industry. At present, industrial robots are constantly progressing in terms of deep learning, cross-border integration, artificial intelligence, etc. The new business forms and models will eliminate some backward production capacity, increase the effectiveness of resource distribution and optimize the structure of the whole industrial chain [24].

Third, from the standpoint of a country, the improvement in the overall robot density can play the "catfish effect", eliminating backward production capacity, more reasonably allocating production factors to enterprises that actively integrate new technologies and have strong independent innovation capabilities, expanding the market share of high-quality enterprises, bringing into play the "size effect," and improving the overall allocation efficiency and total factor productivity of a country. Industrial robots increase the complexity of the industry’s export products and the percentage of domestic added value, enhance the export trade advantages and international competitiveness, and ultimately improve the country’s GVC.

Based on the theoretical analysis above, we propose the first research hypothesis:

Hypothesis 1: Industrial robot applications can improve manufacturing GVC.

### 3.2 Transmission mechanism of highly skilled human capital

Human capital is the fundamental driving force for technological progress and sustainable economic growth. It not only directly affects the output of enterprises as a production factor but is also the dominator of other production factors. Physical capital needs human capital to play its role and generate value. The upgrading of human capital drives technological innovation, increases the income of other physical production factors through knowledge spillover, creates high-added value at low cost, and improves production efficiency and trade competitiveness, which is one of the critical factors in the dynamic evolution of each country’s long-term comparative advantages and status in the international division of labor. While replacing low-skilled labor, industrial robots increase the demand for highly skilled human capital, such as R&D personnel and operators with appropriate specialized skills, because highly skilled human capital is the direct inventor and user of industrial robots and the carrier of new technology creation, integration, and application.

The stage of tasks that industrial robots can replace in the production process is determined by the level of new technology in the industry [25]. New technology requires new education. Suppose the education system does not develop with the new technology. In that case, a mismatch would arise between the technical skills required by new technology and the technical skills possessed by workers, which would hinder the effect of industrial robots and the improvement in the country’s TFP. Highly skilled human capital, as an advanced production factor, can effectively promote the integration of industrial robots into existing production processes and technological routes and maximize the utility of industrial robots.

At present, industrial robots are mainly produced by Fanuc of Japan, Yaskawa of Japan, ABB of Switzerland, and KUKA of Germany. Most countries rely on imports. In this context, industrial robot application promotes an increase in the demand for highly skilled human capital. Through learning by doing, highly skilled human capital can absorb, integrate, and accumulate cutting-edge technology, leading to progressive innovation and achieving breakthrough innovation in specific fields. This will help importing countries realize the transformation from introduction to independent research, enhance the more profound and higher gradient development of domestic industrial robots, and lead to an increase in manufacturing GVC.

Based on the theoretical analysis above, we propose the second research hypothesis:

Hypothesis 2: The application of industrial robots can effectively enhance highly skilled human capital, thereby improving the status of countries in manufacturing GVC.

### 3.3 Transmission mechanism of the producer service industry

The trend of servitization of manufacturing has become increasingly evident in recent years and has become an essential factor in promoting the rationalizing and upgrading of manufacturing. One of the critical connotations of the development of the servitization of manufacturing is the servitization of inputs, that is, the increase in producer service factor inputs in the production process. Producer services include R&D and design, financial credit, postsales services, etc., which are high value-added links and have a higher proportion of domestic value-added than finished products. Manufacturing, which is in the intermediate low value-added segment, can move to the productive services sector at both ends of the value chain only by exploiting the forward and backward effects. Producer services embed information technology, financing credit, and human capital into manufacturing. They can improve the overall value-added level of the industry through the knowledge technology spillover effect.

In addition, industrial robots are inseparable from producer services. Industrial robots rely on the industrial internet, 3D printing, and cloud computing platforms to transform the traditional demand for tangible physical elements into the demand for intangible service elements represented by data and information. In this process, technological patents, R&D, and other producer service inputs can help enterprises integrate industrial robots into new technologies, processes, and concepts and promote product upgrading. Investment in producer services, such as communication networks and management consulting, can reduce high transaction costs and blind production due to information asymmetries, grant companies timely access to the latest industry trends and product demand, introduce market-oriented research and development of specific types of industrial robots, and achieve targeted customer lock-in matching and product recognition improvement. The development of financial credit, business leasing, and other producer service input factors can reduce enterprises’ financing costs and liquidity constraints and improve financial security when introducing industrial robots. The development of consulting accounting, advertising logistics, and distribution alliances enables enterprises to optimize the spatial layout of the supply chain through the professional division of cooperation and industrial system collaboration and focus more on the investment and development of intelligent industrial robots’ core technology, represented by industrial robots, to achieve differentiated competitive advantages.

Industrial robots improve labor productivity, increase the demand for complementary nonproductive tasks, and expand the scale of relevant industries. The development of the production service industry can also absorb workers replaced by industrial robots. The increase in employment can reduce the supply price of production service factors and improve the quality of supply, which can promote the utility of industrial robots to a greater extent.

Based on the theoretical analysis above, we propose the third research hypothesis:

Hypothesis 3: The application of industrial robots can effectively improve the development of productive service industries, thereby enhancing the status of countries in manufacturing GVC.

## 4. Model and data

### 4.1 Model setting

We construct the following econometric model:

$$GVCPosijt=\alpha_{0}+\alpha_{1}{ROD}_{ijt}+\gamma Controls_{it}+\nu_{i}+\nu_{j}+\nu_{t}+\varepsilon_{ijt}$$ (1)

Where the subscript *i*、*j* and *t* denote country, industry and year, respectively. The dependent variable *GVCP* denotes the status of GVC. The core independent variable *ROD* denotes industrial robot density. *ν*_*i*_, *ν*_*j*_ and *ν*_*t*_ denote the fixed effect of country, industry, and year, respectively. *ε*_*ijt*_ denotes the error item.

### 4.2 Indicator construction and data description

#### 4.2.1 Dependent variable

The UIBE GVC Database used the global input‒output model to decompose the trade flows of each country into 16 indicators, providing an accurate and reasonable variable basis for accounting for the relative status of GVC. [26, 27] The specific calculation method is:

GVCP=PLv/PLy (2)

where *PLv* and *PLy* denote the average forward GVC production length and the average backward GVC production length, respectively. They are obtained from the weighted average of the production length of the pure domestic value chain, the traditional trade value chain, and the production length of the simple and complex GVC. The more significant the average forward GVC production length is, the longer the value chain involved in the initial domestic input to the final foreign output is, which means that the status of the GVC of initial domestic input is more upstream. The more significant the average backward GVC production length is, the longer the value chain involved in the initial foreign input to the final domestic output is, which means that the status of GVC of the final domestic output is further downstream. The index *GVCP* is determined by the ratio of the average production length of the forward and backward GVC. A larger value of *GVCP* indicates that the country is more upstream in the GVC. The index *GVCP* data come from the UIBE GVC Indicators database.

#### 4.2.2 Independent variable

The industrial robot density (*ROD*) is equal to the stock of industrial robots divided by the number of employees, where the data of industrial robots are obtained from the IFR database. The calculation method of IFR for the stock of industrial robots assumes that industrial robots do not depreciate in the first 12 years and that they lose all economic value when they are scrapped in the 13th year. We believed that gradual depreciation might be more realistic, so we calculated the industrial robot stock according to the perpetual inventory method, starting from 1993 and assuming that the average annual depreciation rate is 10% [18]. We used the IFR robot inventory measurement method (ROBIFR) and the IFR robot stock measure method (ROBIFR) as robustness tests. Then, we matched the industry classification of the IFR with the industry classification and employment data in WIOD 2016 to calculate the industrial robot density by industry segment for each country in each year. Finally, considering that the presence of too few industrial robot installations prevents them from having a significant economic impact, we removed countries with fewer than 50 cumulative installations of industrial robots since 1993. Considering the unique characteristics of the Hong Kong SAR of China and Chinese Taiwan, we removed the Hong Kong SAR of China, Chinese Taiwan, and other countries that could not be matched. We finally obtained the industrial robot density data of 18 industries in 38 countries.

#### 4.2.3 Mechanism variables

*Highly skilled human capital (HHC)*. We measured the supply of highly skilled human capital by the proportion of the total number of R&D technicians to the country’s total labor force. The R&D technicians have not only specific technical knowledge but also the corresponding production experience and can put new inventions into practice in a timely and smooth manner, which best fits the characteristics of highly skilled human capital complementary to industrial robots. Data on the number of R&D technicians and the total labor force were obtained from the WDI database.

*Producer Services (PSE)*. For the definition of specific industries included in producer services, we used the Statistical Classification of producer services (2019) of the Chinese National Bureau of Statistics. The current measurement methods regarding the development level of producer services are mainly divided into the technological sophistication of imports and exports and the complete consumption coefficient based on the input‒output table, considering that the complete consumption coefficient measures the number of producer services factor inputs in the manufacturing production process, rather than the producer service industry’s development level. Moreover, the analysis of the complete consumption coefficient cannot effectively measure the change in quality of the producer service. The larger the complete consumption coefficient of the producer service is, the greater the quantity of producer services needed. However, the higher the quality of the producer service is, the less consumption input is needed. Therefore, it is contradictory to measure the development level of producer services based on the complete consumption coefficient.

Finally, we used export technological sophistication to measure the development level of producer services. The higher the export technological sophistication is, the stronger the competitiveness of the whole producer services industry and the higher the level of development. The specific measurement method follows the classic method [28], which replaces the traditional trade flows with the domestic added value of exports and calculates the technological sophistication of a specific type of producer services by weighting:

$$PRODY_{j}=\sum \frac{VX_{ji}/\sum_{j}VX_{ji}}{\sum (VX_{ji}/\sum_{j}X_{ji})}Y_{i}$$ (3)

where *PRODY* denotes the export technological sophistication, *VX* denotes the domestic added value of exports, and *Y* is the GDP per capita based on purchasing power parity (PPP). Then, we calculate the export technological sophistication of producer services for each country:

$$EXPY_{i}=\sum_{j}\left(\frac{VX_{ji}}{\sum_{j}VX_{ji}}\right)PRODY_{j}$$ (4)

The data on the added value of exports come from the WIOT, and the data on GDP per capita based on PPP come from the WDI database.

#### 4.2.4 Control variables

*(1) Employment to population ratio*, *15+*, *total (EPR)*. The labor supply situation and demographic structure are key factors influencing the status of countries in manufacturing GVC. Adequate labor security and a reasonable employment rate are essential driving forces for the rise of GVC status.

*(2) GDP per capita (PGDP)*. The PGDP can effectively reflect the level of economic development and has a significant impact on the status of countries in GVC.

*(3) Tariff rate*, *applied*, *weighted mean*, *all products (TAF)*. Different tariff levels across countries can directly impact business trade decisions and the status of countries in GVC.

*(4) Real interest rate (REX)*. Different interest rate levels reflect changes in national fiscal policy and affect the difficulty of enterprise market financing and credit, which can affect the expansion of the enterprise’s trade and production levels, ultimately affecting the status of countries in GVC.

*(5) Economic freedom index (IEF)*. Countries with a higher level of marketization and more complementary economic activities tend to have higher efficiency in resource allocation and can maintain long-term stable development. This indicator is also a good reflection of each country’s business environment.

*(6) Number of employees (EMP)*. Considering that the number of employees in the industry affects the supply price of labor factors for enterprise production, the scale of employees in different industries can also reflect the local scale of the industry in the country, thus affecting the status of countries in the GVC. Therefore, we use the number of employees in each industry in each country to measure the labor reserve and the size of the local industry.

*(7) Export (EXP) and import (IMP) scale*. The size of the industry’s import and export scale reflects its degree of openness to the outside world and the degree of embedding in the international value chain. The changes in import and export scale also indirectly translate the industry’s global value chain position. Therefore, we use the total exports and imports of each country and industry in the same year to measure the scale of industry foreign trade.

Among the above control variables, the data on the economic freedom index are obtained from the Fraser Institute database, the number of employees in each country and industry are obtained from the International Labor Organization, the export and import scale of each country and industry are obtained from the World Trade Database, and the data of other variables are obtained from the WDI database. All indicators are taken as logarithms in the process of empirical analysis. Due to the limited availability of the research data, the sample period is from 2000 to 2014. The main reason is that the core dependent variable, the data on the status of countries in GVC, is derived from the UIBE GVC Database, which is a reliable and widely cited database and recommended by the WTO. However, the most recent data are from 2014, so the research sample period is from 2000 to 2014. Although the time may not be sensitive enough, the data of 18 industries in 38 countries from 2000 to 2014 are sufficiently large panel data, which can effectively support our research. Table 1 shows the descriptive statistics.

**Table 1:** Descriptive statistics.

Variables	Number	Average	Standard	Minimal	Maximum
*GVCPos*	10260	1.356	0.327	0.000	4.425
*ROD*	10260	0.828	1.404	0.000	11.71
*THE*	10260	1.462	0.214	1.063	1.731
*PSE*	10260	7.823	2.061	4.691	13.59
*EPR*	10260	4.018	0.120	3.664	4.328
*PGDP*	10260	9.941	0.975	6.719	11.42
*TAF*	10260	0.506	0.674	0.000	2.519
*REX*	10260	4.586	0.117	4.008	4.928
*IEF*	10260	2.129	0.0830	1.828	2.274
*EMP*	10260	4.166	1.648	0.693	7.128
*EXP*	10260	8.145	1.542	5.678	10.79
*IMP*	10260	7.608	0.766	6.600	9.807

## 5. Results

### 5.1 Benchmark

We use the fixed effect model to explore the impact of industrial robot applications on the status of countries in manufacturing GVC. First, we examine the direct effect without adding control variables, and then we gradually add control variables. The benchmark regression results are shown in Table 2.

**Table 2:** Benchmark regression results.

Variables	Model 1	Model 2	Model 3	Model 4
	*GVCP*	*GVCP*	*GVCP*	*GVCP*
*ROD*	0.0044**	0.0059***	0.0059***	0.0052***
	(2.2300)	(2.9929)	(2.9675)	(2.6070)
*EPR*		0.0352	0.0453	0.0673**
		(1.0565)	(1.3577)	(2.0753)
*PGDP*		-0.0202	-0.0063	0.0451
		(-1.2814)	(-0.3000)	(1.5698)
*TAF*		-0.0088	-0.0112*	-0.0103*
		(-1.4384)	(-1.7829)	(-1.7185)
*REX*			-0.0480***	-0.0331***
			(-4.0679)	(-2.6210)
*IEF*			0.0122	0.0314
			(0.2343)	(0.6131)
*EMP*				-0.0174*
				(-1.7430)
*EXP*				-0.0057
				(-1.2816)
*IMP*				-0.0213**
				(-2.3561)
Constant	1.3521***	1.4146***	1.4309***	1.0039***
	(831.5222)	(7.9092)	(8.0416)	(4.3523)
National	YES	YES	YES	YES
Industry	YES	YES	YES	YES
Year	YES	YES	YES	YES
N	10260	10260	10260	10260
R2	0.9764	0.9765	0.9766	0.9768

Note: In brackets is the corresponding t value of the estimated result

***, **, * represent statistical significance at 1%, 5%, and 10%, respectively.

From the regression results, we know that the estimated coefficient of industrial robot density is significantly positive, indicating that industrial robot application can effectively improve the status of countries in manufacturing GVC. After adding other control variables and fixed effects, the estimated coefficient of industrial robot density is still significantly positive, which further verifies Hypothesis 1.

### 5.2 Robustness

To ensure that the conclusions are robust and reliable, we conduct robustness tests from the following aspects:

First, considering the different accounting methods for the industrial robot stock, we use the IFR industrial robot stock calculation method to calculate the industrial robot density. The results are shown in Model 1 of Table 3.

**Table 3:** Robustness test results.

Variables	Model 1	Model 2	Model 3	Model 4	Model 5
	*GVCP*	*APL*	*RCA*	*GVCPat*	*MEXPY*
*ROD*		0.0051***	0.0165***	0.0057*	0.0071***
		(2.5940)	(4.2511)	(1.8577)	(3.6220)
*RODIFR*	0.0054***				
	(2.9798)				
*EPR*	0.0705**	0.0671**	-0.1046	-0.1211**	0.0295
	(2.1715)	(2.0717)	(-1.3996)	(-1.9801)	(0.6627)
*PGDP*	0.0429	0.0451	0.0002	0.0602	-0.1168***
	(1.4867)	(1.5689)	(0.0046)	(1.2441)	(-6.8708)
*TAF*	-0.0103*	-0.0102*	-0.0415***	-0.0369***	-0.0433***
	(-1.7010)	(-1.7122)	(-3.5627)	(-3.7410)	(-7.6782)
*REX*	-0.0347***	-0.0326***	-0.1212***	0.0788***	0.0878***
	(-2.7433)	(-2.5907)	(-3.7866)	(2.8341)	(7.3676)
*IEF*	0.0344	0.0310	0.2512**	0.2004*	-0.2446***
	(0.6775)	(0.6065)	(2.0520)	(1.8976)	(-5.3362)
*EMP*	-0.0177*	-0.0175*	0.1773***	-0.0009	0.0135
	(-1.7769)	(-1.7578)	(9.6192)	(-0.0506)	(1.3767)
*EXP*	-0.0060	-0.0056	0.0147	-0.0251**	0.0049
	(-1.3427)	(-1.2615)	(1.3429)	(-2.1866)	(0.8831)
*IMP*	-0.0208**	-0.0213**	-0.0230	-0.0237*	0.0319***
	(-2.2787)	(-2.3568)	(-1.4916)	(-1.7210)	(4.3687)
*Constant*	1.0127***	1.0117***	0.5364	0.2599	484.1383***
	(4.3644)	(4.3884)	(1.1261)	(0.6174)	(1,879.1267)
National	YES	YES	YES	YES	YES
Industry	YES	YES	YES	YES	YES
Year	YES	YES	YES	YES	YES
N	10260	10260	10260	10260	10260
R^2^	0.9768	0.9742	0.9587	0.9395	0.9735

Second, the Antràs and Fally Upstream Index (*APL*) is a relatively mature index that can reflect the embedded position of GVC and thus the value-added capability of each industry. Therefore, we use the Antràs and Fally Upstream Index as alternative indicators of the GVC for regression, and the results are shown in Model 2 of Table 3. Antràs and Fally Upstream Index data are obtained from the UIBE GVC Indicators database.

Third, the index of revealed comparative advantage (*RCA*) is a well-established indicator that responds to the strength and changing trend of product competitiveness, so we use it as a substitute indicator for GVC. Moreover, we construct the RCA index based on the benchmark of trade value-added data [26] and construct the RCA index based on trade value-added data [27], and the results are shown in Model 3 of Table 3.

Fourth, the more countries participate in GVC, the more likely they are to benefit from GVC. More extensive GVC participation (*GVCPat*) tends to be positively correlated with the status of countries in GVC, so we use it as an alternative indicator of GVC status. The GVCPat is calculated by the ratio of the forward and backward participation indexes of GVC. A high GVCPat value indicates higher GVC participation and higher GVC status. The results are shown in Model 4 of Table 3.

Fifth, improving the country’s overall export technological sophistication can effectively exert spillover effects and synergistic effects and improve the value chains of other domestic industries. Therefore, we construct the country’s overall export technological sophistication (*MEXPY*) as the explained variable, and the results are shown in Model 5 of Table 3.

After replacing the industrial robot stock measurement method in Model 1, its regression coefficient remains positive. The estimated value has not changed significantly, indicating that the conclusion that the increase in industrial robot density can improve the GVC status does not change because of the different measurement methods of industrial robot stock. After replacing the dependent variable with the Antràs and Fally Upstream Index in Model 2, the regression coefficient of industrial robot density is still significantly positive, indicating that industrial robot application can improve labor productivity and help countries climb up the value chain. The results in Model 3 show that the increase in industrial robot density can improve the relative competitive advantage of products and increase the value added of products. The regression results in Model 4 show that the rise in the density of industrial robots makes manufacturing products more competitive and increases exports. Moreover, the expansion of the industrial scale triggers an increase in the import demand for intermediate inputs and deepens the trade exchanges between countries. The estimated results in Model 5 show that industrial robot applications can enhance countries’ overall export technological sophistication. The increase in robot density in various industries within countries can have complementary effects and industrial cluster advantages and jointly promote a country’s overall competitiveness.

### 5.3 Endogeneity test

Considering that the use of industrial robots can promote a country’s upgrading of GVC and that the enhanced GVC status can enable the country to obtain more trade income and funds to purchase more industrial robots, the bilateral causality between the increase in industrial robot density and the upgrade in GVC status may cause an endogeneity problem. For this reason, we use the time lag of first-order and second-order variables of industrial robot density as instrumental variables and use the 2SLS method for regression. We also use the same method and setting to regress the Antràs and Fally Upstream Index (APL) to ensure robustness and effectiveness. The estimation results are shown in Model 1 and Model 2 of Table 4.

**Table 4:** Endogenous treatment.

Variables	Model 1	Model 2	Model 3	Model 4	Model 5	Model 6
	*GVCP*	*APL*	*GVCP*	*GVCP*	*APL*	*APL*
*L*.*GVCP*			0.9565***	0.9586***		
			(21.6302)	(25.2422)		
*L*.*APL*					0.9566***	0.9581***
					(21.6245)	(25.0446)
*ROD*	0.0058***	0.0058***	0.0075**	0.0056**	0.0076**	0.0057**
	(6.3188)	(6.3031)	(2.0533)	(2.3650)	(2.1009)	(2.4420)
*EPR*	0.0325*	0.0324*	-0.0495	-0.0351	-0.0515	-0.0353
	(1.7964)	(1.7928)	(-0.5535)	(-0.5413)	(-0.5834)	(-0.5591)
*PGDP*	0.0449***	0.0449***	-0.0147	-0.0110	-0.0145	-0.0108
	(3.3571)	(3.3595)	(-1.0019)	(-1.0660)	(-0.9484)	(-1.0980)
*TAF*	-0.0123***	-0.0123***	-0.0264	-0.0139	-0.0252	-0.0127
	(-3.6530)	(-3.6530)	(-1.4022)	(-1.1560)	(-1.2781)	(-1.0909)
*REX*	-0.0369***	-0.0366***	-0.0544	-0.0244	-0.0528	-0.0232
	(-5.3424)	(-5.2973)	(-0.7783)	(-0.6236)	(-0.7509)	(-0.5934)
*IEF*	0.0268	0.0255	0.0008	0.0227	-0.0012	0.0230
	(0.9174)	(0.8710)	(0.0091)	(0.3310)	(-0.0146)	(0.3229)
*EMP*	-0.0158***	-0.0159***	0.0105	0.0082	0.0101	0.0076
	(-3.4025)	(-3.4355)	(1.1772)	(1.3229)	(1.0716)	(1.1535)
*EXP*	-0.0049**	-0.0048**	0.0021	0.0025	0.0019	0.0019
	(-2.2273)	(-2.2058)	(0.3376)	(0.5348)	(0.3129)	(0.4089)
*IMP*	-0.0223***	-0.0223***	0.0170	0.0096	0.0172	0.0099
	(-5.0319)	(-5.0383)	(0.7407)	(0.5736)	(0.7660)	(0.5610)
*Constant*			0.4632	0.2446	0.4667	0.2423
			(0.8858)	(0.7341)	(0.8951)	(0.7612)
National	YES	YES	YES	YES	YES	YES
Industry	YES	YES	YES	YES	YES	YES
Year	YES	YES	YES	YES	YES	YES
Kleibergen-Paap rk LM		584.925 [0.000]				
Cragg-Donald Wald F		1.6e+04 [19.93]				
Kleibergen-Paap rk Wald F		567.104 [19.93]				
Hansen J test		4.595 [0.0321]				
AR (1) P			0.000	0.000	0.000	0.000
AR (2) P			0.388	0.324	0.372	0.310
Hansen P			0.463	0.463	0.525	0.525
N	8892	8892	9576	9576	9576	9576
R^2^	0.0213	0.0213				

Notes: P-values are reported in square brackets [] below the Kleibergen-Paap rk LM test and Hansen J test, and critical values at the Stock-Yogo 10% level are reported in square brackets [] below the Cragg-Donald Wald F test and Kleibergen-Paap rk Wald F test.

Considering that there is a certain inertia and lag in the change in GVC status, the existence of missing variables may lead to endogeneity. Therefore, in this paper, the time lag of the first order of the dependent variables is included to establish a dynamic model, which is carried out using system GMM. System GMM includes a one-step method and a two-step method. The two-step system GMM standard covariance matrix is steadier. However, it can easily cause a significant underestimation of the standard deviation of the parameter estimates when the sample is small. Although the efficiency of the one-step system GMM is relatively low, it is consistent. To ensure the robustness of the conclusions, we report the regression results of both one-step and two-step system GMM and regress the Antràs and Fally Upstream Index (APL) with the same method. The estimation results are shown in Model 3 to Model 6 of Table 4.

According to the results in Model 1 and Model 2 of Table 4, we know that the instrumental variables selected are reasonable and adequate. On this basis, the estimated coefficients of industrial robot density based on 2SLS are significantly positive regardless of whether the dependent variables are GVCP or APL, indicating that the increased industrial robot density enhancement can effectively improve the GVC status in the absence of possible endogenous bilateral causality. As shown in Model 3 to Model 6 of Table 4, the time lags of the first-order terms of GVCP and APL are significantly positive, reflecting that the change in value chain position has a certain continuity and that climbing up the value chain is a slow and smooth development process. In addition, the industrial robot density in Model 3 to Model 6 of Table 4 is positive and significant, indicating that the rise in industrial robot density still has explanatory power for GVC status improvement when the omitted variables and lagged effects of the dependent variables are excluded.

### 5.4 Heterogeneity

Considering that the research sample in this paper contains multiple countries and industries, the unique attributes of different countries and industries may have a heterogeneous impact on the improvement effect on GVC status. To investigate this heterogeneous effect, we divide developed and developing countries based on the World Bank classification criteria and conduct the regression by grouping. The results are shown in Table 5. Moreover, based on the use characteristics of factors [29], we divide industries into labor-intensive, capital-intensive, and technology-intensive industries, and the results are shown in Model 3 to Model 5 of Table 5.

**Table 5:** Heterogeneity analysis.

Variables	Model 1	Model 2	Model 3	Model 4	Model 5
	Developed	Developing	Labour	Capital	Technology
	*GVCP*	*GVCP*	*GVCP*	*GVCP*	*GVCP*
*ROD*	0.0030***	0.0106***	0.0114**	0.0061**	0.0093**
	(3.4411)	(4.4353)	(2.0541)	(2.1579)	(2.5380)
*EPR*	0.1512***	-0.4664***	0.0363	0.1329**	0.0659
	(21.6618)	(-28.2624)	(0.7691)	(2.1148)	(1.2546)
*PGDP*	-0.1449***	0.0885***	0.0847*	0.0179	-0.0575
	(-19.0894)	(13.5542)	(1.8742)	(0.3524)	(-0.9410)
*TAF*	-0.0080***	-0.0858***	-0.0243**	-0.0130*	0.0148
	(-10.3175)	(-47.0292)	(-2.0494)	(-1.8468)	(1.6324)
*REX*	0.0998***	0.0415***	-0.0496***	-0.0320	-0.0041
	(48.0133)	(10.6391)	(-2.7652)	(-1.5223)	(-0.1469)
*IEF*	-0.3549***	-0.3556***	-0.0315	0.0778	0.1766
	(-65.6668)	(-37.9169)	(-0.4904)	(0.8569)	(1.3778)
*EMP*	-0.0028	0.0181	-0.0214*	-0.0038	0.0029
	(-0.3894)	(1.3865)	(-1.7353)	(-0.3400)	(0.1166)
*EXP*	0.0068	0.0026	-0.0033	-0.0150	-0.0144
	(1.3414)	(0.3650)	(-0.5490)	(-1.5583)	(-1.5616)
*IMP*	-0.0135***	0.0725***	-0.0239	-0.0215	-0.0026
	(-3.8767)	(22.3191)	(-1.5169)	(-1.4624)	(-0.1993)
*Constant*	484.0551***	484.3732***	0.9036**	1.0688***	1.1789***
	(9,566.0490)	(13,940.5804)	(2.3850)	(2.7055)	(2.6392)
National	YES	YES	YES	YES	YES
Industry	YES	YES	YES	YES	YES
Year	YES	YES	YES	YES	YES
N	3,780	6,480	4,560	3,420	2,280
R^2^	0.942	0.969	0.152	0.207	0.075

From the results in Model 1 and Model 2 of Table 5, we know that although the estimated coefficients of industrial robot density in both the developed and developing country subgroups are significantly positive, the absolute value of the estimated coefficients in the developing country subgroup is more significant than that in the developed country subgroup. This indicates that the impact of industrial robots is more effective in developing countries, and industrial robot application can more effectively promote developing countries to achieve technological catch-up and reduce the development gap. The application of industrial robots in developing countries can reduce labor costs, reduce labor risks, improve product quality, support the learning and absorption of advanced international technologies, and enhance their own scientific and technological research capabilities through technological cooperation with developed countries, giving rise to the advantage of backwardness and thus more quickly upgrading their GVC status [6].

From the results in Model 3 to Model 5 of Table 5, we can see that the absolute values of the estimated coefficients of industrial robot density are larger and more significant in the labor-intensive and technology-intensive subgroups. The reason may be that industrial robots replace traditional low-skilled labor, improve labor efficiency, reveal comparative advantage, and enhance the output level and value added of labor-intensive industries. Moreover, the use of industrial robots brings technological upgrading, which enables enterprises to optimize and upgrade their products. Industrial robots are the core component of Industry 4.0, which combines intelligent sensors with industrial equipment to improve productivity, enhance reliability, and reduce operating costs. Industrial robots can also collect and analyze data about themselves and adjust production progress to ensure maximum production efficiency, allowing robots to manage and coordinate production [15]. Therefore, industrial robot application is more effective in improving the GVC of labor-intensive and technology-intensive industries.

### 5.5 Mechanism

To clarify the specific mechanism of the impact of industrial robot applications to improve the status of countries in GVC, we use the method of interaction items to test the mechanism effect. The interaction items and the core independent variable are included in the regression equation. The results of the mechanism test are shown in Table 6.

**Table 6:** Mechanism test.

Variables	Model 1	Model 2	Model 3	Model 4
	*GVCP*	*GVCP*	*GVCP*	*GVCP*
*ROD*		-0.0004		-0.0093
		(-0.1161)		(-1.2231)
*HHC*	0.0078***	0.0052		
	(3.1052)	(1.7084)		
*ROD×HHC*		0.0039**		
		(1.8154)		
*PSE*			0.0143**	0.0117
			(1.8196)	(1.4721)
*ROD×PSE*				0.0013**
				(1.9669)
*EPR*	0.0514*	0.0701**	0.0882**	0.1075***
	(1.8389)	(2.4550)	(2.4440)	(2.9088)
*PGDP*	0.0644*	0.0399	0.0539	0.0137
	(2.1000)	(1.2590)	(1.5471)	(0.3244)
*TAF*	-0.0080	-0.0100	-0.0141	-0.0172*
	(-1.1972)	(-1.5032)	(-1.5899)	(-2.0238)
*REX*	-0.0336***	-0.0378***	-0.0254**	-0.0281**
	(-3.1759)	(-3.3833)	(-2.4680)	(-2.6491)
*IEF*	0.0040	0.0293	-0.0099	0.0261
	(0.0885)	(0.6585)	(-0.2039)	(0.4847)
*EMP*	-0.0168	-0.0173	-0.0197	-0.0187
	(-1.4068)	(-1.5020)	(-1.5114)	(-1.4621)
*EXP*	-0.0055	-0.0057	-0.0055	-0.0053
	(-0.9961)	(-1.0160)	(-0.9944)	(-0.9955)
*IMP*	-0.0237***	-0.0197**	-0.0272***	-0.0204**
	(-3.2095)	(-2.5959)	(-4.1476)	(-2.8823)
*Constant*	0.9423***	1.0505***	0.8328***	1.0525***
	(3.8775)	(4.2838)	(3.9262)	(4.3036)
National	YES	YES	YES	YES
Industry	YES	YES	YES	YES
Year	YES	YES	YES	YES
N	10,260	10,260	10,260	10,260
R^2^	0.977	0.977	0.977	0.977

From the results in Model 1 to Model 3 of Table 6, we can see that the estimated coefficients of highly skilled human capital (*HHC*) are all significantly positive, indicating that highly skilled human capital itself has a promoting effect on the value chain status and upstream degree. When highly skilled human capital interacts with robot density, the interaction coefficient is significantly positive, indicating that industrial robot applications will not replace employment for highly skilled humans but will promote their role and increase the demand for highly skilled humans. Humans and machines synergistically complement each other, enabling machines to have a greater status upgrading effect on GVC. The enhancement of highly skilled human capital plays a vital transmission mechanism. Thus, Hypothesis 2 is confirmed.

The results in Model 4 to Model 6 of Table 6 show that the regression coefficients of productive services (*PSE*) are significantly positive, which means that the higher the development level of producer services, the stronger the international competitiveness of the country’s export products, the higher the value added included, and thus the more upstream a country is in GVC. When the productive service interacts with robot density, the coefficient of the interaction term is significantly positive, indicating that the increased density of industrial robots promotes the integration and absorption of producer service factors and enhances the competitiveness of enterprises. In addition, the rise of industrial robot density ensures that industrial robots can match with more advanced and efficient service factors and obtain and apply the latest technology in a timely manner to enhance production efficiency and competitiveness. Improving the development level of productive services becomes a critical transmission mechanism. Thus, Hypothesis 3 is confirmed.

## 6. Extension

The previous analysis verified that the increased density of industrial robots could improve the GVC status of the manufacturing industry. However, since countries with large-scale industrial robots are mainly developed countries or developing countries with sound economic performance, many countries with limited economic development that have not yet adopted industrial robots on a large scale are also participating in the GVC cycle. Therefore, is the impact of industrial robot adoption on value chain positions a real advancement in the country’s value chain position, or is it simply a "passive" advancement in countries that have adopted industrial robots because the countries that have not yet adopted industrial robots on a large scale are "falling behind"?

To analyze this issue, we first calculate the gap between the GVC location index, the upstream degree index, and the highest value in each industry in each country, producing new variables GVCPD and APLD as the dependent variables. Then, we replace industrial robot density with its value of one period delayed as the core explanatory variable and hold other control variables unchanged to explore the impact of industrial robot applications on the gap between value chain positions. In addition, we construct a "limited catch-up index" based on export technological sophistication [30]. First, we calculate each country’s actual export technological sophistication each year, represented by ln*EXPY*_*it*_, and then fit the export technological sophistication based on the GDP per capita. We use $({lnEXPY)}_{it}^{f}$ to represent the fitted value of the export technological sophistication; then, the limited catch-up index *(LCI*) is defined as

$$LCI_{it}=lnEXPY_{it}-({lnEXPY)}_{it}^{f}$$ (5)

If *LCI*>0, it means that the country has fully used its advantages and achieved a breakthrough and limited catch-up. If *LCI*≦0, the country does not give full play to its comparative advantage, and its GVC status is not improved. The regression results are shown in Table 7.

**Table 7:** Value chain gap degree and limited catch-up index.

Variables	Model 1	Model 2	Model 3	Model 4	Model 5	Model 6
	*GVCP*	*GVCP*	*GVCP*	*GVCP*	*GVCP*	*GVCP*
*ROD*					0.0029**	0.0071***
					(2.7417)	(7.2214)
*L*.*ROD*	-0.0274***	-0.0313***	-0.0280***	-0.0318***		
	(-3.5141)	(-4.1228)	(-3.5708)	(-4.1811)		
*EPR*		-0.0229		-0.0291		0.0295***
		(-0.1566)		(-0.1980)		(3.3029)
*PGDP*		0.0710		0.0742		-0.0887***
		(0.6158)		(0.6424)		(-13.2470)
*TAF*		0.0172		0.0165		-0.0433***
		(0.7076)		(0.6792)		(-47.2796)
*REX*		0.0712		0.0701		0.0878***
		(0.7925)		(0.7796)		(46.9651)
*IEF*		-0.2950		-0.2929		-0.2446***
		(-1.0134)		(-1.0049)		(-32.9654)
*EMP*		-0.1151***		-0.1152***		0.0135
		(-3.6502)		(-3.6398)		(1.5401)
*EXP*		0.1303***		0.1300***		0.0049
		(4.5235)		(4.5065)		(0.9808)
*IMP*		-0.0272		-0.0290		0.0319***
		(-0.6605)		(-0.7021)		(12.3728)
*Constant*	0.8939***	0.1970	0.8985***	0.2120	-0.0024**	0.5586***
	(145.3172)	(0.2229)	(145.5216)	(0.2393)	(-2.7417)	(19.6795)
National	YES	YES	YES	YES	YES	YES
Industry	YES	YES	YES	YES	YES	YES
Year	YES	YES	YES	YES	YES	YES
N	9,576	9,576	9,576	9,576	10,260	10,260
R^2^	0.790	0.793	0.792	0.795	0.973	0.975

From the results in Model 1 to Model 4 of Table 7, we know that the estimated coefficient of the one-period delayed term of industrial robot density is significantly negative regardless of whether the dependent variable is the value chain position index or the upstream degree index and whether control variables are included in the regression equation. This indicates that the increase in industrial robot density can effectively narrow the gap between value chain positions among countries, and industrial robots absolutely promote countries’ status in GVC.

The regression coefficients of industrial robot density to the limited catch-up index in Model 5 and Model 6 of Table 7 are positive and significant, which implies that industrial robot application can help a country fully exploit its comparative advantage and achieve limited catch-up in GVC status.

## 7. Conclusions and policy recommendations

In the era of intelligent manufacturing, industrial robots have become a focus of investment in various countries. However, their impact on GVC status has not been effectively tested and clarified. This paper constructs the industrial robot density index at the manufacturing level. It explores the impact of industrial robot application on the status of countries in GVC and its mechanism. The study found that, first, industrial robot application can increase the value added of manufacturing, enhance export technological sophistication and improve the status of countries in GVC. Second, the heterogeneity analysis shows that GVC upgrading effects are more pronounced in developing countries and labor-intensive and technology-intensive industries. Third, industrial robot application creates new value by reintegrating highly skilled human capital in new ways, making the role of highly skilled human capital more important. Moreover, in the context of manufacturing servitization, industrial robot application improves the development level and supply quality of producer services. Highly skilled human capital and producer services represent a critical transmission mechanism. Finally, the extended analysis shows that industrial robot applications can narrow the gap in manufacturing value chain positions between countries and help countries give full play to their comparative advantages and achieve limited catch-up.

The research findings provide the following policy inspiration for governments’ future development plans. (1) Increase industrial robot applications, promote the in-depth integration of industrial robots, and obtain more excellent value added through intelligent manufacturing. (2) Strengthen investment in the education of scientific and technological R&D personnel, train more high-skilled professionals with core technology and innovative ability and maximize the utility of industrial robots. (3) Grasp the trend of manufacturing services, improve the technological advancement of domestic production services, actively absorb and introduce international advanced technology patents, and help domestic enterprises transform and upgrade through knowledge spillover and market competition.

Due to data availability, author ability and other reasons, this paper still has some limitations. For example, due to limitations in the timeliness of data available regarding the global value chain of the manufacturing industry, our data may not be time-sensitive enough. In terms of the research object, we conducted our analysis at the macro industry level but could not delve into the micro enterprise level. In terms of the control and mediating variables, the indicators chosen may not be sufficiently comprehensive because of the difficulty of obtaining complete data at the cross-national industry level. Future research can explore the relationship between industrial robots and the status of countries in countries in manufacturing global value chains at the enterprise level based on inputs and outputs and explore the influence mechanism from a more comprehensive perspective.
